# Prefectural difference in spontaneous intracerebral hemorrhage incidence in Japan analyzed with publically accessible diagnosis procedure combination data: possibilities and limitations

**DOI:** 10.4178/epih.e2016028

**Published:** 2016-07-02

**Authors:** Toru Fukuhara, Yusuke Hori

**Affiliations:** Department of Neurological Surgery, National Hospital Organization Okayama Medical Center, Okayama, Japan

**Keywords:** Database, Incidence, Mortality, Cerebral hemorrhage

## Abstract

**OBJECTIVES:**

Annually reported, publically accessible Diagnosis Procedure Combination (DPC) data from the Japanese government is a part of the total DPC database of the Japanese medical reimbursement system for hospitalization. Although medical issues can be evaluated with these data promptly, the applicability of these data in epidemiological analyses has not been assessed.

**METHODS:**

We performed analyses using only statistical indices reported on the a government website. As a preliminary step, the prefectural consistency of spontaneous intracerebral hemorrhage (sICH) was examined with prefectural mortality over 20 years. Then the prefectural incidence of sICH for four years was calculated, utilizing publically accessible DPC data. To determine its reliability, the consistency was examined, and correlations were analyzed with three prefectural factors expected to have an effect: the elderly rate, mortality due to sICH, and the non-DPC bed rate. In addition, a comparison model between prefectures with this method was developed by analyzing other prefecture-specific factors.

**RESULTS:**

Prefectural mortality due to sICH and prefectural sICH incidence in the DPC database were both consistent over the years. Prefectural sICH incidence had a constant positive correlation with the elderly rate, a partial correlation with mortality due to sICH, but no correlation with the non-DPC bed rate, which is one of the major biases when utilizing the DPC database. In the comparison model, the factors of low income and alcohol consumption showed increased sICH incidence.

**CONCLUSIONS:**

Although careful attention to its limitations is required, publically accessible DPC data will provide insights into epidemiological issues.

## INTRODUCTION

Although many reports have estimated spontaneous intracerebral hemorrhage (sICH) incidence in certain regions [1,2], no nationwide survey capturing all sICH patients has been reported. To obtain the exact sICH incidence, all newly diagnosed sICH cases in the population with available baseline information must be captured, which is difficult even in a small community, and any reported sICH incidence should be regarded as involving some error. Although higher age [1] and male [1,3] increases sICH incidence, even after adjusting for these factors, large regional differences are likely to exist [2]. Hence, estimated sICH incidences in certain regions should not be interpreted as having universal meaning. These estimations would have significance if they were comparable among different regions.

The recent expansion of electronic medical records has enabled large amounts of information to be prepared for medical analysis [4]. In Japan, a novel medical reimbursement system for hospitalization, named the Diagnosis Procedure Combination (DPC) system, was launched in 2003, based on electronic medical records from physicians, submitted by hospitals participating in this system to the Japan Ministry of Health, Labor, and Welfare (JMHLW) [5]. These data are analyzed by the government and the summarized data are officially reported once a year. The number of hospitals submitting their hospitalized patient information to the DPC system (DPC hospitals) has gradually increased over 10 years, and as of March 2015, among 894,216 general beds for admission in Japan, 582,367 beds have been covered by the DPC system, which is 65.1% of the total. The DPC system is believed to cover approximately 90% of the total number of acute inpatient hospitalizations [6,7], as most active hospitals are now DPC hospitals. Since the information from DPC hospitals directly relates to the reimbursement of hospitalization expenses, the data are carefully checked at each hospital, and the submitted information is investigated by the government. The platform format of submissions is uniform throughout the nation, and the data to be submitted includes: age, sex, primary diagnosis, consciousness status and comorbidities on admission, all interventions performed during admission, and activity status at discharge [8]. We believe the quality of the DPC database is quite high in spite of its size.

**Figure 1.**
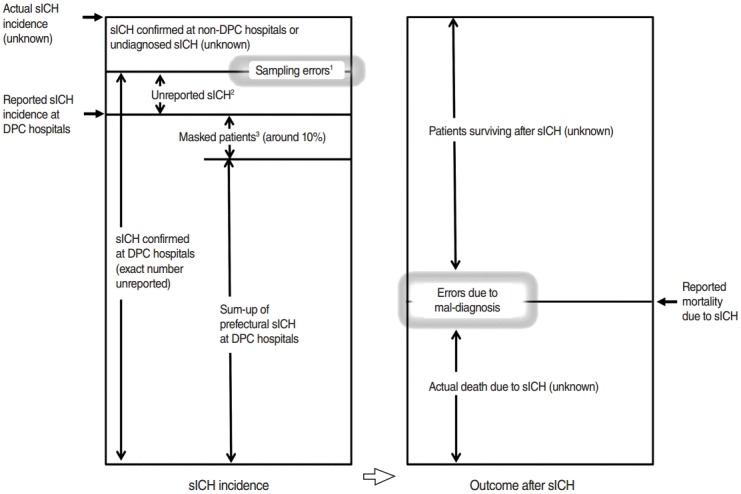
Conceptional diagram indicating the relation between sICH incidence and mortality due to sICH. sICH, spontaneous intracerebral hemorrhage; DPC, Diagnosis Procedure Combination. ^1^Sampling errors in obtaining patients in the DPC database include misdiagnosis and inappropriate coding or double counts of the same patients. ^2^Unreported sICH patients from publically accessible DPC data include patients who died or were discharged within 24 hours. ^3^Masked patients are unreported patients treated at hospitals with fewer than 10 patients in a year. Specifically, for sICH patients, patients with ICH due to arteriovenous malformation rupture will be involved due to the lack of corresponding codes.

However, in utilizing publically accessible DPC data, careful attention should be paid to the limitations of the data [5]. As illustrated in Figure 1, the actual sICH incidence in Japan is unknown because of uncaptured sICH patients at hospitals that do not submit DPC data (non-DPC hospitals). Although the number of sICHs at DPC hospitals is known by JMHLW, patients who died within 24 hours are not reported to the public; thus the number of sICHs that can be gathered from publically accessible DPC data is smaller than the number contained by the whole DPC database. In publically accessible DPC data, numbers fewer than 10 in the segmentalized fields of each hospital according to the DPC codes are masked, although the total patient numbers of DPC hospitals are reported. When obtaining prefectural patient numbers by summing up the patient numbers of the hospitals in the same prefectures, this masking will result in a further reduction in the number of patients compared with the total patient number in the DPC database [2].

Considering these limitations, whether publically accessible DPC data is worth analyzing has yet to be determined, although its enormity, its accessibility, and its expected further development are attractive. The purpose of this paper was to examine the adequacy of utilizing publically accessible DPC data in epidemiological fields.

## MATERIALS AND METHODS

This study required no institutional review board approval, or informed consent, since only publically accessible data on government homepages obtained through the Internet were used.

### Prefectural consistency in spontaneous intracerebral hemorrhage mortality

Prior to examining the adequacy of prefectural sICH incidence in the DPC database, whether sICH occurs with a constant tendency in each prefecture should be examined, since randomly fluctuating medical events are inappropriate for epidemiological analysis. Although there has been no comparable prefectural sICH incidence measurement so far, as a related index, the number of deaths due to sICH was utilized for this purpose. The Japanese government has successively reported the total annual number of deaths categorized by cause. This number is obtained from all of the death certificates physicians produced, which are then regrouped by cause of death according to the International Classificstion of Diseas, 10th revision by the government. Although mortality and incidence are different indices, it can be assumed that there are correlations, and as a preliminary analysis, the prefectural consistency concerning sICH was confirmed utilizing this officially reported mortality. Every five years, the government reported the rate of age-adjusted cause of death for each prefecture by sex (Appendix 1A). If death due to sICH is affected by surrounding conditions specific to each prefecture, the rank of this rate must be highly constant and the tendencies must be close between sexes. We statistically analyzed the prefectural consistency of sICH mortality between 1990 and 2010 and the correlation between the tendencies in both sexes was evaluated for each year.

### Obtaining spontaneous intracerebral hemorrhage incidence in the Diagnosis Procedure Combination database

From publically accessible DPC data, the number of hospitalized patients due to sICH in DPC hospitals can be obtained. The corresponding codes indicating sICH are those beginning with 010040, and in this article, the number of patients with those codes divided by the population of the corresponding year was defined as the sICH incidence in the DPC database. However, this number was contaminated by two inevitable biases: one is that patients who died within the first day were excluded from the publically accessible DPC data, and the other is that intracerebral hemorrhages (ICH) due to arteriovenous malformation rupture were counted as sICH, although ICH due to aneurysm rupture, tumor, cerebral infarction, or trauma with different corresponding codes were ruled out. As for the prefectural sICH incidence, hospitals were grouped according to the prefecture in which they were located, then the sICH patient number for each prefecture was summed up. This number divided by the prefectural population is defined as the prefectural sICH incidence; however, this number is further contaminated by the “masked rate” [5]. This occurs because patient numbers treated in hospitals with fewer than 10 patients in a year were not reported, thus the patient numbers obtained as the prefectural sICH incidence resulted in smaller numbers than those captured by the DPC database. This “masked rate,” the percentage of unreported numbers in Excel files giving the patient numbers of each hospital, was obtained by a 100× summation / total number. All calculated sICH incidences were expressed as the number per 100,000 people. The files used for the calculation are shown in Appendix 1B and 1C.

### Evaluation of prefectural spontaneous intracerebral hemorrhage incidence in the Diagnosis Procedure Combination database

In order to evaluate the adequacy of the prefectural sICH incidence in the DPC database, obtained with the abovementioned method, as an epidemiologically applicable index, analyses were performed concerning two factors: its consistency over a number of years and its correlation with other factors that might have an effect. The consistency of the prefectural sICH incidence in the DPC database between 2011 and 2014 was analyzed according to the same manner used in the consistency analysis for prefectural mortality. Then, the correlations with three possibly affecting factors were assessed for each year. The first possibly affecting factor is the prefectural rate of elderly population aged 75 and over, each year of which was obtained from the files shown in Appendix 1C. A large elderly population will increase the sICH incidence, and without a correlation with this factor, the adequacy of the obtained prefectural sICH would be doubtful. As a second factor, the correlation with the prefectural crude mortality due to sICH was evaluated, since mortality due to sICH is a major outcome. The crude prefectural sICH mortality was calculated by dividing total reported deaths due to sICH in each prefecture, with the files shown in Appendix 1D, by the total prefectural population in each corresponding year (Appendix 1C). The third factor is a major, considerable bias: the rate of non-DPC beds in each prefecture. As mentioned above, the prefectural sICH incidence may be contaminated by several biases, and the most significant bias would be the unknown number of sICH patients treated at non-DPC hospitals. If prefectures with a larger number of non-DPC general beds tend to have smaller sICH incidences, the effect of this bias may contaminate the analysis, thus the prefectural sICH incidence obtained with this method should be considered unreliable. The numbers of DPC beds in each hospital were obtained from the files shown in Appendix 1E, and the total number of general hospital beds in each prefecture from the files shown in Appendix 1F, for the calculation of the prefectural rate of non-DPC general beds.

### Comparison model between prefectural sICH incidences in the DPC database

Although the purpose of this article is to evaluate the adequacy of prefectural sICH incidence in the DPC database as an epidemiological index, it is also important to understand how this can be utilized in further analysis. In order to demonstrate a comparison model as an example, the latest prefectural sICH incidence in the DPC database in 2014 was analyzed with several factors specific to each prefecture. The factors used for analysis were 1) population per 1 km^2^ of inhabitable area, 2) yearly temperature average, 3) yearly hours of sunshine, 4) yearly precipitation, 5) prefectural income per person, and 6) prefectural alcohol consumption per adult person. These factors were obtained from the government database indicated in Appendix 1G and 1H.

### Statistical analysis

The analysis for the time-course consistency of prefectures was performed by calculating Kendall’s coefficient of concordance [9,10]. For the correlation, the nonparametric Spearman’s correlation coefficient was used for the univariate analysis, and multiple forward stepwise regression analysis was employed for the multivariate analysis. The results were considered statistically significant at p<0.05, and all p-values were two-sided.

## RESULTS

### Prefectural consistency for spontaneous intracerebral hemorrhage mortality

The age-adjusted mortality due to sICH throughout Japan decreased from 26.1 (per 100,000 people) in 1990 to 17.1 in 2010 for males, and from 15.7 to 7.6 for females. There was a large difference among prefectures; for males from 11.1 to 24.6 in 2010, and for females from 4.8 to 12.1. During these 20 years, five prefectures were continuously ranked among the lowest 10 prefectures for males; Nara (ranked 5-2-1-2-1), Kagawa (ranked 2-9-4-5-1), Shiga (ranked 7-5-8-3-3), Osaka (ranked 10-8-9-7-8), and Fukui (ranked 1-3-2-10-10), and for females, two prefectures were ranked; Kagawa (ranked 2-1-7-5-1) and Osaka (ranked 6-5-5-6-3). As for the highest 10 prefectures, five prefectures were continuously ranked for males, Iwate (ranked 2-1-2-1-1), Aomori (ranked 4-6-1-4-3), Akita (ranked 8-3-7-6-4), Kagoshima (ranked 6-5-5-2-5), and Tochigi (ranked 1-2-8-8-7), but none for females. In Kendall’s coefficient of concordance analysis, both males (W-value=0.75, p<0.001) and females (W-value=0.70, p<0.001) showed a high consistency of prefectural sICH mortality. There are strong correlations between sexes, Rs=0.68, p<0.001 in 1990, Rs=0.52, p<0.001 in 1995, Rs=0.69, p< 0.001 in 2000, Rs=0.77, p<0.001 in 2005, and Rs=0.77, p<0.001 in 2010. These consistencies over time and between sexes revealed that there should exist a prefectural tendency in mortality due to sICH. All data are shown in Appendix 2.

### Overview of spontaneous intracerebral hemorrhage incidence in the Diagnosis Procedure Combination database

Table 1 presents an overview of sICH incidence in the DPC database. The crude sICH incidence in the DPC database increases from 43.6 to 47.3 in these four years, which must result from the increasing number of hospitals using DPC systems. The “masked rate” of summed-up sICH patients for each prefecture is around 10%; thus the prefectural sICH number in the DPC database obtained with the above mentioned method captured approximately 90% of patients from the total sICH number in the DPC database.

### Consistency of prefectural spontaneous intracerebral hemorrhage in the Diagnosis Procedure Combination database

During these four years, four prefectures were continuously ranked among the lowest 10 prefectures; Yamanashi (ranked 1-1-1-1), Miyagi (ranked 2-3-2-3), Oita (ranked 3-2-4-9), and Saitama (ranked 6-4-3-2). As for the highest 10 prefectures, eight prefectures were continuously ranked, Okinawa (ranked 1-1-1-1), Iwate (ranked 2-3-2-2), Wakayama (ranked 3-4-5-4), Kumamoto (ranked 4-5-6-8), Yamagata (ranked 5-2-3-7), Tottori (ranked 6-10-8-3), Kochi (ranked 7-6-7-5), and Aomori (ranked 10-9-9-9). The Kendall’s coefficient of concordance analysis revealed quite a high consistency of prefectural sICH incidence in the DPC database for these four years (W-value=0.95, p<0.001). All data were shown in Appendix 3.

### Factors affecting prefectural spontaneous intracerebral hemorrhage incidence in the DPC database

**Figure 2.**
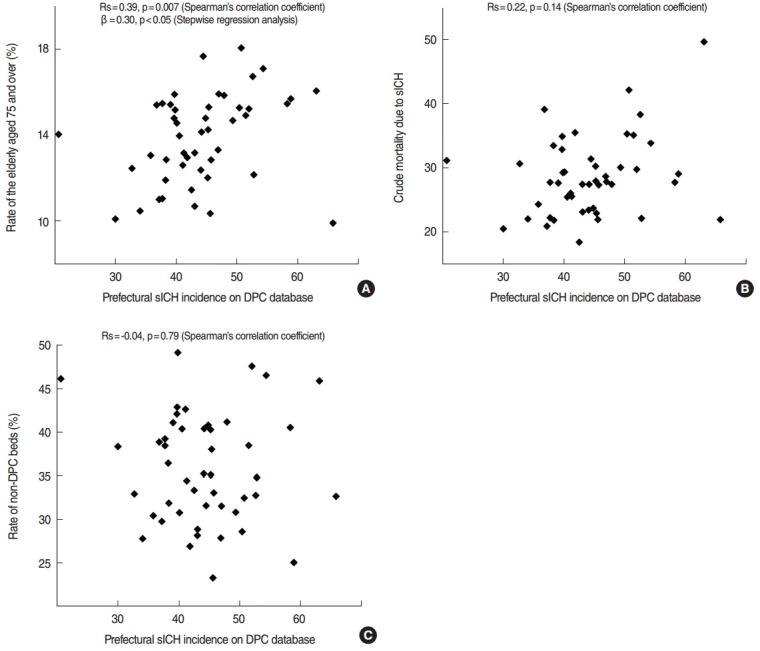
Scattergrams indicating the relationship with prefectural sICH incidence in the DPC database in 2014. (A) The rate of the elderly aged 75 and over among prefectures varied from 0.099 to 0.180, and positive correlations were observed in both univariate and multivariate analyses. (B) The crude mortality due to sICH among prefectures varied from 18.5 to 49.7, and positive but insignificant correlation was observed. (C) The rate of non-DPC beds among prefectures varied from 0.23 to 0.49, and negative but insignificant correlation was observed. sICH, spontaneous intracerebral hemorrhage; DPC, Diagnosis Procedure Combination.

Table 2 indicates the Spearman’s correlation coefficient of three examined factors in each year, and Figure 2 indicates the relations between sICH incidence and these factors in 2014. Among examined factors, only the rate of the elderly aged 75 and over showed a significant positive correlation continuously for these four years. The crude mortality due to sICH showed positive correlation and the rate of non-DPC beds showed negative correlation, but they were non-significant. With a multiple regression analysis, no factor significantly affected the prefectural sICH in the DPC database in 2011 and in 2012; however, in 2013, the crude mortality due to sICH had a significant correlation (β=0.37, p=0.01), and in 2014, the rate of elderly persons had a significant correlation (β=0.30, p<0.05). All data from the three examined factors are shown in Appendices 4-6.

### Comparison model between prefectural s spontaneous intracerebral hemorrhage incidences in the Diagnosis Procedure Combination database

**Figure 3.**
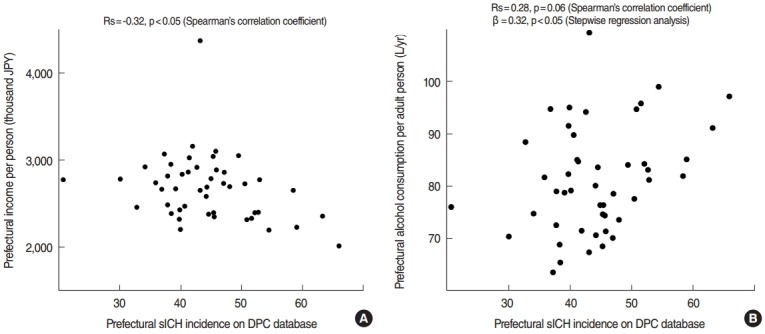
Scattergrams indicating the relationship with prefectural incidence in the DPC database in 2014 as the comparison model. Among examined factors, those with a significant correlation are shown. (A) Prefectural income per person varied from 2,018 thousand to 4,373 thousand JPY, and negative correlation was observed in univariate analysis. (B) Prefectural alcohol consumption per adult person varied from 63.5 to 109.4 L/yr, and positive correlation was observed in multivariate analysis. sICH, spontaneous intracerebral hemorrhage; DPC, Diagnosis Procedure Combination; JPY, Japanese yen (100 JPY=1 dollar).

Among six factors examined, only the prefectural income per person negatively correlated with prefectural sICH incidence according to the non-parametric Spearman’s correlation coefficient (Rs=-0.32, p<0.05). With multivariate analysis, the prefectural alcohol consumption per adult person independently affected prefectural sICH incidence (β=0.32, p<0.05). Other factors did not reveal any significant relation in both analyses. Figure 3 indicates the relations between sICH incidence and these significant factors. All data are shown in Appendix 7.

## DISCUSSION

The reliability of a large medical database has been difficult to determine. Ordinarily, comparison with a “gold standard” database is desirable for novel database evaluation. However, there is no “gold standard” database regarding sICH incidence [2]. Among medical indices relating to sICH, the standard one is mortality due to sICH, which can be extracted from the vital statistics survey reported annually by the JMHLW, and these data have been used for international comparison by the World Health Organization [11]. In our preliminary analysis, mortality due to sICH in Japan revealed a constant tendency among prefectures. However, our study also revealed only partial positive tendencies between the prefectural mortality and prefectural sICH incidence in the DPC database. This does not necessarily mean that the sICH incidence in the DPC database is unreliable, since the mortality is a different parameter from the incidence, as shown in Figure 1. The mortality due to sICH was reported to be lower in Japan than in any other region [1], and this might result from an intensive medical intervention attitude even for comatose patients with sICH [12]. Further, we should be skeptical about the accuracy of the reported mortality number, since this also definitely contains errors due to misdiagnosis [13,14].

Several population-based studies for sICH incidence in Japan have been reported, all of which were from regional communities [3,15-17]. The Japanese government holds a National Database (NDB) composed of all health insurance claims (HIC) for payment, which covers the entire nation, including non-DPC hospitals [18]. Although NDB has been refined with more computerized HIC submissions [19], NDB is not equally adequate to the DPC database for analysis due to the lack of detailed patient profiles or uncoded diagnoses [18,19]. We showed constant positive correlations for prefectural sICH incidence in the DPC database with the elderly population rate for each prefecture, indicating a rational tendency: a more aged population will have more sICHs [1]. No significant correlation was obtained with the rate of non-DPC beds in each prefecture, which is a considerable bias. These positive and negative results, and some correlations with mortality due to sICH, may suggest some potential for the sICH incidence obtained by this method. When considering the reliability of the database, its extensive coverage of all concerned patients is a necessary factor to take into account. Due to the severity of the symptoms, it is very rare for patients with sICH to be treated without hospitalization [20]. Thus, analysis of hospitalized patient data is effective for sICH research [20], and was actually not so different from analysis with a population-based study [21]. As a hospital patient database, the DPC database has been expanding and enhancing its coverage. In addition, the credibility of the gathered data is important for the reliability of the database. We believe the quality of the recorded data is high due to its relation to reimbursement under government supervision.

Although there are attractive features to the DPC database, we should be careful regarding limitations when utilizing the DPC database. The major limitation is that the number of patients treated at non-DPC hospitals is decreasing as the medical reimbursement for hospitalization using the DPC system expands. However, when utilizing publically accessible DPC data, as in this article, other specific limitations occur. The limitations inherent to the publically accessible DPC data are significant and this may account for the paucity of articles utilizing these data [5,22]. Analyses with the whole body of the DPC database are able to utilize more detailed information for each patient. In order to avoid the limitations inherent to publically accessible DPC data, it is preferable that the whole body of the DPC database to be made accessible to any qualified researcher with a medically meaningful research proposal.

Since determining the causative factors influencing sICH incidence is not the purpose of this article, we performed only correlation analysis with a limited number of factors, in order to introduce a comparison model between prefectures using the latest publically accessible DPC data. Among the factors examined in this model, the prefectural income per person negatively correlated with the prefectural sICH incidence, and prefectural alcohol consumption was a factor with a positive effect. The risk of alcohol consumption has been proven in Japan [17], and considering the correlation of sICH incidence with low income, some social behaviors relating to drinking habits may influence sICH onset [23]. Other meteorological factors did not reveal any significant relation with sICH incidence, although several reports indicate meteorological [15] or seasonal effects [24,25] on sICH onset. Further analyses with reliable data representing the characteristics of each prefecture may show causative factors with this comparison model.

Although careful attention due to its limitations is required, further evaluation of publically accessible DPC data in Japan as a tool for epidemiological analyses is warranted.

## Figures and Tables

**Table 1.** Annual change in the DPC database related to sICH

Year (report date)	No. of hospitals in DPC database	Rate of DPC beds (DPC beds/all general beds, %)	Total sICH	sICH DPC database^1^	Summed-up incidence in sICH	Masked rate^2^ (%) prefectural
2011 (21/08/2012)	1,634	54.6 (491, 282/899, 385)	55,766	43.6	49,941	10.4
2012 (09/30/2013)	1,774	57.2 (513, 449/898, 166)	55,889	43.8	50,195	10.2
2013 (09/05/2014)	1,804	57.6 (516, 551/897, 380)	56,486	44.4	50,747	10.2
2014 (11/16/2015)	2,942	65.1 (582, 367/894, 216)	60,144	47.3	53,817	10.5

DPC, Diagnosis Procedure Combination; sICH, spontaneous intracerebral hemorrhage.

1 Numbers represent patients per 100,000 Japanese people.

2 Masked rate results from unreported patients treated at hospitals with fewer than 10 patients per year.

**Table 2.** Correlation coefficient of analyzed factors with prefectural sICH incidence in the DPC database

Year	Analyzed factor		
	Rate of the elderly aged 75 and over	Crude mortality due to sICH	Rate of non-DPC beds
2011	0.35 (0.02)	0.25 (0.10)	-0.24 (0.11)
2012	0.36 (0.01)	0.28 (0.06)	-0.21 (0.17)
2013	0.37 (0.01)	0.27 (0.07)	-0.24 (0.11)
2014	0.39 (0.007)	0.22 (0.14)	-0.04 (0.79)

The number in the parenthesis represents p-value. sICH, spontaneous intracerebral hemorrhage; DPC, Diagnosis Procedure Combination.

## Appendix 1.

The details of publically accessible data on government homepages obtained through the Internet

## Appendix 2.

Age-adjusted prefectural mortality due to sICH between 1990 and 2010 (per 100,000 people)

## Appendix 3.

Prefectural sICH incidence on the DPC database (per 100,000 people)

## Appendix 4.

Rate of the elderly aged 75 and over

## Appendix 5.

Crude mortality due to sICH (per 100,000 people)

## Appendix 6.

Rate of non-DPC beds

## Appendix 7.

Prefectural data of examined factors in comparison model with prefectural sICH incidence on the DPC database in 2014, shown in Appendix 3
