# Supplementary material for: Prefectural difference in spontaneous intracerebral hemorrhage incidence in Japan analyzed with publically accessible diagnosis procedure combination data: possibilities and limitations
Source: Epidemiol Health. 2016 Jul 2;38:e2016028. doi: 10.4178/epih.e2016028 (PMC5037357; doi:10.4178/epih.e2016028)
Supplement: Supplementary file 1 [file epih-38-e2016028-app1.pdf]

Appendix 1. The details of publically accessible data on government homepages obtained through the Internet

A. The age-adjusted numbers of death due to sICH per each prefecture in 1990, 1995, 2000, 2005 and 2010 were obtained from the CSV files on e-Stat (Japanese government statistics portal site), indicated as Table 2-31 at, https://www.e-stat.go.jp/SG1/estat/GL08020103.do?_toGL08020103_&listID=000001101037&requestSender=search. Accessed December 12, 2015.

B. The numbers of sICH on the DPC database were obtained from the Excel files on one of JMHLW home pages, reporting the conference series evaluating the DPC system at, http://www.mhlw.go.jp/stf/shingi/shingi-chuo.html?tid=128164. The total numbers of sICH in Japan were obtained from the Excel files, indicated as Reference 2-(9), in which the hospitals were grouped by the year of participation in the DPC system at, http://www.mhlw.go.jp/stf/shingi/2r9852000002hs9l-att/2r9852000002hsv2.xls for 2011 (reported on August 21, 2012), http://www.mhlw.go.jp/file/05-Shingikai-12404000-Hokenkyoku-Iryouka/0000034285.xls for 2012 (reported on September 20, 2013), http://www.mhlw.go.jp/file/05-Shingikai-12404000-Hokenkyoku-Iryouka/0000056548.xls for 2013 (reported on September 5, 2014) and http://www.mhlw.go.jp/file/05-Shingikai-12404000-Hokenkyoku-Iryouka/0000104212.xls for 2014 (reported on November 16, 2015). The prefectural numbers of sICH were obtained from the Excel files, indicated as Reference 2-(9) MDC01 at, http://www.mhlw.go.jp/stf/shingi/2r9852000002hs9l-att/2r9852000002hsv9.xls for 2011, http://www.mhlw.go.jp/file/05-Shingikai-12404000-Hokenkyoku-Iryouka/0000023588.xls for 2012, http://www.mhlw.go.jp/file/05-Shingikai-12404000-Hokenkyoku-Iryouka/0000056549.xls for 2013 and http://www.mhlw.go.jp/file/05-Shingikai-12404000-Hokenkyoku-Iryouka/0000104214.xls for 2014. All accessed December 12, 2015.

C. The population in Japan in each prefecture and the numbers of people aged 75 and over were obtained from the Excel file on e-Stat, indicated as Table 11 at, http://www.e-stat.go.jp/SG1/estat/List.do?lid=000001088119 for 2011, http://www.e-stat.go.jp/SG1/estat/List.do?lid=000001109855 for 2012, http://www.e-stat.go.jp/SG1/estat/List.do?lid=000001118081 for 2013 and http://www.e-stat.go.jp/SG1/estat/List.do?lid=000001132435 for 2014. All accessed December 12, 2015.

D. The crude numbers of deaths due to sICH per each prefecture were obtained from the CSV files on e-Stat, indicated as Table 5-19 at, http://www.e-stat.go.jp/SG1/estat/List.do?lid=000001101884 for 2011, http://www.e-stat.go.jp/SG1/estat/List.do?lid=000001108739 for 2012, http://www.e-stat.go.jp/SG1/estat/List.do?lid=000001108740 for 2013 and http://www.e-stat.go.jp/SG1/estat/List.do?lid=000001137965 for 2014. All accessed December 12, 2015.

E. The bed numbers of each prefecture on the DPC system were obtained from the Excel files on one of the JMHLW homepages, reporting the conference series evaluating the DPC system at, http://www.mhlw.go.jp/stf/shingi/shingi-chuo.html?tid=128164. The files were indicated as "the overview of participating hospitals" at, http://www.mhlw.go.jp/stf/shingi/2r9852000002hs9l-att/2r9852000002i3fr.xls for 2011, http://www.mhlw.go.jp/file/05-Shingikai-12404000-Hokenkyoku-Iryouka/0000023535.xls for 2012, http://www.mhlw.go.jp/file/05-Shingikai-12404000-Hokenkyoku-Iryouka/0000056493.xls for 2013 and http://www.mhlw.go.jp/file/05-Shingikai-12404000-Hokenkyoku-Iryouka/0000104287.xls for 2014. All accessed December 12, 2015.

F. The numbers of general beds per each prefecture were obtained from the CVS files on e-Stat, indicated as G10 at, http://www.e-stat.go.jp/SG1/estat/List.do?lid=000001102729 for 2011, indicated as G9 at, http://www.e-stat.go.jp/SG1/estat/List.do?lid=000001112557 for 2012 http://www.e-stat.go.jp/SG1/estat/List.do?lid=000001126654 for 2013 and http://www.e-stat.go.jp/SG1/estat/List.do?lid=000001141081 for 2014. All accessed December 12, 2015.

G. The statistical indices of Japanese social life were annually reported by the Ministry of Internal Affairs and Communications and the latest one was reported on February 19, 2015 at, http://www.e-stat.go.jp/SG1/estat/GL08020103.do?_toGL08020103_&tclassID=000001056523&cycleCode=0&requestSender=estat. The population per 1 km2 of inhabitable area was obtained from the Excel file, indicated as "Table A-Population and Households" at, http://www.e-stat.go.jp/SG1/estat/images/xls.gif;jsessionid=NwYfWkzW3TXpT544Slm9p3tnjktbcFKXJ4f2nlTH2RqwL1m3Dq9K!-352016752!1540013709 (2013 as the latest data), yearly average of temperature, yearly sunshine hours and yearly precipitation were obtained from the Excel file, indicated as "Table B-Natural Environment" at, http://www.e-stat.go.jp/SG1/estat/images/xls.gif;jsessionid=NwYfWkzW3TXpT544Slm9p3tnjktbcFKXJ4f2nlTH2RqwL1m3Dq9K!-352016752!1540013709 (2013 as the latest data) and prefectural income per person was obtained from the Excel file, indicated as "Table C-Economic Base" at, http://www.e-stat.go.jp/SG1/estat/images/xls.gif;jsessionid=NwYfWkzW3TXpT544Slm9p3tnjktbcFKXJ4f2nlTH2RqwL1m3Dq9K!-352016752!1540013709 (2011 as the latest data). All accessed December 12, 2015.

H. The total amount of alcohol consumption was obtained from the Excel file on the homepage of the National Tax Agency at https://www.nta.go.jp/kohyo/tokei/kokuzeicho/jikeiretsu/xls/13.xls. (2013 as the latest data). Each prefectural amount of alcohol consumption was divided by the number of people aged 20 and over in each prefecture, obtained from the Excel file, indicated as Table 10 at, http://www.e-stat.go.jp/SG1/estat/List.do?lid=000001118081. All accessed December 12, 2015.
