# Supplementary material for: Prefectural difference in spontaneous intracerebral hemorrhage incidence in Japan analyzed with publically accessible diagnosis procedure combination data: possibilities and limitations
Source: Epidemiol Health. 2016 Jul 2;38:e2016028. doi: 10.4178/epih.e2016028 (PMC5037357; doi:10.4178/epih.e2016028)
Supplement: Supplementary file 2 [file epih-38-e2016028-app2.pdf]

Appendix 2. Age-adjusted prefectural mortality due to sICH between 1990 and 2010 (per 100,000 people)

Year	1990	1995	2000	2005	2010
Male					
National average	26.1	25.0	20.3	19.0	17.1
Name of Prefecture					
Hokkaido	20.5	22.6	19.2	19.3	15.7
Aomori	33.6	28.6	28.8	24.1	23.0
Iwate	34.0	32.5	26.8	25.7	24.6
Miyagi	26.3	26.5	20.7	23.4	23.3
Akita	30.0	29.8	24.1	22.8	22.9
Yamagata	24.5	25.5	19.6	18.8	19.5
Fukushima	30.4	29.3	19.7	22.1	17.9
Ibaraki	31.6	27.0	22.6	21.9	20.0
Tochigi	34.7	30.0	23.8	22.5	20.7
Gumma	29.1	23.7	21.4	18.9	17.2
Saitama	27.9	27.8	21.4	19.2	16.2
Chiba	25.9	25.1	20.9	19.3	15.8
Tokyo	28.5	25.5	20.2	18.9	18.4
Kanagawa	27.4	24.8	21.9	20.1	17.4
Niigata	22.7	25.9	19.9	20.4	19.8
Toyama	21.0	19.5	15.5	16.1	16.1
Ishikawa	21.6	15.5	15.2	16.2	12.5
Fukui	18.6	17.3	14.1	16.1	14.5
Yamanashi	25.1	26.2	14.8	15.5	15.9
Nagano	29.1	28.6	24.2	20.7	17.2
Gifu	24.8	24.1	20.3	18.2	16.3
Shizuoka	26.4	23.8	22.9	22.7	19.9
Aichi	23.6	25.5	21.6	19.4	17.0
Mie	26.4	24.5	18.3	17.8	15.8
Shiga	22.0	20.2	16.0	13.4	12.3
Kyoto	25.6	25.8	19.3	21.0	14.7
Osaka	22.5	21.3	16.9	15.7	14.1
Hyogo	24.2	23.0	17.5	15.7	16.0
Nara	21.4	16.9	13.5	12.4	11.1
Wakayama	25.4	22.0	17.7	12.2	13.1
Tottori	33.8	26.8	20.7	22.0	16.4
Shimane	25.5	21.2	18.9	14.4	15.7
Okayama	24.4	20.5	16.9	17.3	18.3
Hiroshima	23.0	26.5	20.3	17.3	16.4
Yamaguchi	24.9	23.4	22.1	18.9	14.7
Tokushima	22.4	28.0	22.3	16.1	13.0
Kagawa	20.2	21.6	15.1	14.7	11.1
Ehime	22.2	22.8	19.2	19.0	14.2
Kochi	29.5	27.1	25.5	24.2	20.3
Fukuoka	25.7	25.5	20.7	17.3	14.9
Saga	28.7	28.2	15.8	19.6	12.8
Nagasaki	26.1	25.1	18.4	17.4	16.9
Kumamoto	28.1	27.9	17.8	18.2	17.9
Oita	27.0	26.0	20.6	16.0	15.1
Miyazaki	27.5	26.7	19.8	19.4	18.5
Kagoshima	30.9	28.9	25.3	24.7	22.4
Okinawa	23.0	28.4	26.2	21.3	21.9

(continued)

Year	1990	1995	2000	2005	2010
Female					
National average	15.7	14.3	10.8	9.3	7.6
Name of Prefecture					
Hokkaido	12.4	12.1	10.2	9.9	7.4
Aomori	17.2	16.8	11.6	10.8	9.2
Iwate	16.6	16.5	13.7	11.9	12.1
Miyagi	15.2	16.4	11.3	9.3	9.7
Akita	15.6	13.8	12.7	10.9	9.0
Yamagata	14.6	12.0	9.9	9.0	7.7
Fukushima	18.6	15.0	12.7	10.7	7.8
Ibaraki	16.6	16.4	11.8	12.1	9.8
Tochigi	20.4	17.1	13.4	12.1	9.2
Gumma	17.0	16.2	13.8	10.9	6.7
Saitama	16.2	17.2	12.3	9.4	7.8
Chiba	17.2	14.9	10.7	10.1	7.4
Tokyo	17.0	15.5	10.6	9.2	8.3
Kanagawa	16.8	15.8	11.7	9.0	8.1
Niigata	15.1	13.0	11.1	9.5	10.1
Toyama	11.6	11.6	9.3	9.3	6.9
Ishikawa	13.5	12.6	8.1	6.4	5.9
Fukui	13.1	12.0	6.6	8.6	8.1
Yamanashi	16.8	10.5	9.3	7.9	6.7
Nagano	16.2	15.2	13.3	10.3	7.4
Gifu	19.5	14.1	11.0	9.3	8.1
Shizuoka	15.3	15.3	11.1	12.0	9.7
Aichi	17.6	14.8	12.3	10.7	8
Mie	18.5	14.5	9.9	8.4	7.5
Shiga	15.1	12.2	10.1	6.8	7.4
Kyoto	17.0	15.7	11.3	9.1	6.9
Osaka	12.6	11.7	8.6	7.2	5.4
Hyogo	15.5	13.8	10.2	7.6	6.5
Nara	11.1	13.3	8.5	4.5	5.4
Wakayama	12.3	11.1	9.7	7.0	6.5
Tottori	17.7	13.9	12.0	9.4	11.3
Shimane	14.4	14.8	9.6	7.2	5.1
Okayama	14.1	13.4	9.8	8.3	6.7
Hiroshima	15.3	14.9	10.0	9.4	6.7
Yamaguchi	14.1	14.9	9.6	10.6	7.2
Tokushima	16	12.6	10.9	9.0	6.6
Kagawa	11.4	10.5	9.2	7.1	4.8
Ehime	15.4	12.6	8.4	8.0	7.0
Kochi	17.2	16.2	12.4	11.1	8.3
Fukuoka	14.2	12.8	9.4	7.4	6.3
Saga	18.6	15.6	10.5	8.5	6.2
Nagasaki	13.7	13.2	9.6	8.4	6.6
Kumamoto	15.1	12.4	9.8	9.2	8.2
Oita	16.1	12.2	10.5	10.2	5.9
Miyazaki	15.3	13.1	12.5	10.4	7.8
Kagoshima	16.8	18.9	11.9	12.5	9.9
Okinawa	14.6	12.6	8.8	8.1	7.4

sICH, spontaneous intracerebral hemorrhage.
