# Supplementary material for: Prefectural difference in spontaneous intracerebral hemorrhage incidence in Japan analyzed with publically accessible diagnosis procedure combination data: possibilities and limitations
Source: Epidemiol Health. 2016 Jul 2;38:e2016028. doi: 10.4178/epih.e2016028 (PMC5037357; doi:10.4178/epih.e2016028)
Supplement: Supplementary file 3 [file epih-38-e2016028-app3.pdf]

Appendix 3. Prefectural sICH incidence on the DPC database (per 100,000 people)

Year	2011	2012	2013	2014
Name of Prefecture				
Hokkaido	31.63	30.26	34.19	40.56
Aomori	49.82	48.81	52.43	51.48
Iwate	61.34	58.40	61.47	63.08
Miyagi	27.42	27.83	27.06	32.73
Akita	46.79	47.60	55.33	50.72
Yamagata	52.80	59.20	56.70	52.61
Fukushima	38.64	39.30	37.46	39.69
Ibaraki	37.32	36.32	39.75	45.22
Tochigi	38.85	34.99	38.32	38.28
Gumma	41.93	44.88	44.41	45.75
Saitama	29.68	28.20	30.20	30.03
Chiba	31.01	33.38	34.25	37.74
Tokyo	40.34	39.47	40.02	43.08
Kanagawa	30.79	33.09	32.50	34.07
Niigata	33.57	35.41	34.68	36.79
Toyama	51.38	48.98	43.03	49.35
Ishikawa	31.99	30.87	33.65	35.81
Fukui	41.22	39.67	35.85	40.13
Yamanashi	18.44	21.83	20.90	20.69
Nagano	50.19	47.37	48.11	50.40
Gifu	42.78	42.94	42.22	43.07
Shizuoka	44.68	44.02	42.06	41.84
Aichi	41.05	43.91	43.21	45.62
Mie	46.45	46.03	41.41	46.90
Shiga	35.36	35.27	33.55	37.22
Kyoto	29.07	38.74	38.14	41.11
Osaka	37.94	38.05	39.44	42.54
Hyogo	41.42	42.94	42.35	44.09
Nara	29.08	31.87	34.49	38.37
Wakayama	60.60	55.47	53.63	58.29
Tottori	51.62	48.80	52.60	58.89
Shimane	37.50	42.15	45.87	44.48
Okayama	40.60	39.72	41.76	44.18
Hiroshima	42.03	39.15	38.63	41.30
Yamaguchi	49.51	47.66	51.90	47.02
Tokushima	47.44	49.61	48.05	47.91
Kagawa	37.60	32.76	37.16	44.85
Ehime	30.08	31.45	32.03	39.07
Kochi	51.45	51.86	52.62	54.34
Fukuoka	49.10	48.36	50.79	52.80
Saga	42.38	39.50	38.93	45.27
Nagasaki	43.97	46.66	43.09	45.38
Kumamoto	53.50	55.01	53.14	52.01
Oita	28.46	25.82	30.81	37.75
Miyazaki	31.74	32.59	31.16	39.86
Kagoshima	37.79	37.46	36.85	39.75
Okinawa	70.09	67.64	64.24	65.80

sICH, spontaneous intracerebral hemorrhage; DPC, Diagnosis Procedure Combination.

Appendix 4. Rate of the elderly aged 75 and over

Year	2011	2012	2013	2014
Name of Prefecture				
Hokkaido	0.127	0.132	0.136	0.140
Aomori	0.136	0.141	0.147	0.149
Iwate	0.149	0.153	0.158	0.160
Miyagi	0.116	0.120	0.122	0.125
Akita	0.167	0.173	0.178	0.180
Yamagata	0.158	0.162	0.166	0.167
Fukushima	0.140	0.144	0.147	0.148
Ibaraki	0.110	0.114	0.117	0.120
Tochigi	0.112	0.114	0.117	0.119
Gumma	0.120	0.123	0.127	0.129
Saitama	0.087	0.092	0.097	0.101
Chiba	0.096	0.101	0.106	0.111
Tokyo	0.098	0.102	0.105	0.107
Kanagawa	0.093	0.097	0.101	0.105
Niigata	0.146	0.149	0.152	0.154
Toyama	0.140	0.143	0.145	0.147
Ishikawa	0.124	0.127	0.129	0.131
Fukui	0.139	0.143	0.145	0.146
Yamanashi	0.132	0.135	0.138	0.140
Nagano	0.145	0.148	0.151	0.153
Gifu	0.122	0.126	0.129	0.132
Shizuoka	0.119	0.123	0.127	0.130
Aichi	0.093	0.097	0.101	0.104
Mie	0.125	0.128	0.131	0.133
Shiga	0.104	0.107	0.109	0.110
Kyoto	0.116	0.120	0.123	0.126
Osaka	0.101	0.106	0.111	0.115
Hyogo	0.113	0.117	0.121	0.124
Nara	0.116	0.121	0.125	0.129
Wakayama	0.145	0.150	0.152	0.154
Tottori	0.150	0.153	0.156	0.157
Shimane	0.170	0.174	0.177	0.176
Okayama	0.133	0.137	0.139	0.141
Hiroshima	0.123	0.127	0.130	0.132
Yamaguchi	0.150	0.154	0.157	0.159
Tokushima	0.151	0.155	0.157	0.158
Kagawa	0.141	0.145	0.146	0.148
Ehime	0.145	0.149	0.152	0.154
Kochi	0.162	0.166	0.169	0.171
Fukuoka	0.113	0.117	0.119	0.122
Saga	0.137	0.140	0.142	0.143
Nagasaki	0.144	0.148	0.151	0.153
Kumamoto	0.145	0.148	0.150	0.152
Oita	0.146	0.149	0.153	0.155
Miyazaki	0.142	0.147	0.150	0.152
Kagoshima	0.151	0.155	0.157	0.159
Okinawa	0.091	0.094	0.097	0.099
