# Supplementary material for: Prefectural difference in spontaneous intracerebral hemorrhage incidence in Japan analyzed with publically accessible diagnosis procedure combination data: possibilities and limitations
Source: Epidemiol Health. 2016 Jul 2;38:e2016028. doi: 10.4178/epih.e2016028 (PMC5037357; doi:10.4178/epih.e2016028)
Supplement: Supplementary file 5 [file epih-38-e2016028-app5.pdf]

Appendix 5. Crude mortality due to sICH (per 100,000 people)

Year	2011	2012	2013	2014
Name of Prefecture				
Hokkaido	27.3	25.2	24.5	25.5
Aomori	38.8	37.9	36.7	35.1
Iwate	50.8	48.9	48.0	49.7
Miyagi	34.3	32.4	31.5	30.7
Akita	46.8	45.7	46.3	42.1
Yamagata	41.8	36.1	34.7	38.3
Fukushima	32.8	32.1	32.7	32.9
Ibaraki	30.1	31.0	31.6	30.3
Tochigi	34.1	34.8	30.6	33.5
Gumma	27.8	30.4	29.7	27.4
Saitama	22.0	20.8	20.6	20.6
Chiba	21.7	24.7	21.5	22.3
Tokyo	25.0	24.0	23.9	23.2
Kanagawa	23.0	22.2	24.2	22.1
Niigata	39.2	35.1	36.6	39.1
Toyama	32.9	32.1	30.0	30.1
Ishikawa	25.5	25.9	26.9	24.4
Fukui	24.2	30.2	23.2	29.4
Yamanashi	32.3	28.9	27.6	31.2
Nagano	32.7	35.3	36.8	35.3
Gifu	29.6	26.7	25.4	27.5
Shizuoka	32.7	35.4	34.1	35.5
Aichi	23.6	23.1	22.6	22.0
Mie	30.6	28.9	28.2	28.7
Shiga	22.1	22.6	19.3	21.0
Kyoto	23.8	26.4	24.1	26.1
Osaka	19.5	19.6	18.9	18.5
Hyogo	22.9	23.8	24.3	23.5
Nara	20.0	19.1	22.1	21.9
Wakayama	23.8	26.4	25.5	27.8
Tottori	32.8	29.6	31.2	29.1
Shimane	33.9	34.6	34.3	31.4
Okayama	30.0	26.6	30.7	27.5
Hiroshima	26.9	26.6	27.3	25.6
Yamaguchi	29.2	29.5	29.8	27.9
Tokushima	28.2	28.1	26.0	27.5
Kagawa	26.7	25.2	26.1	23.8
Ehime	33.1	28.8	27.9	27.7
Kochi	35.9	37.0	36.4	33.9
Fukuoka	23.4	22.7	21.3	22.2
Saga	26.3	30.1	26.1	28.0
Nagasaki	26.6	27.5	27.2	23.0
Kumamoto	31.1	30.1	26.7	29.8
Oita	27.9	31.0	30.4	27.8
Miyazaki	31.1	33.2	31.6	29.3
Kagoshima	39.3	37.8	36.1	34.9
Okinawa	22.0	21.8	22.3	22.0

sICH, spontaneous intracerebral hemorrhage.

Appendix 6. Rate of non-DPC beds

Year	2011	2012	2013	2014
Name of Prefecture				
Hokkaido	0.531	0.495	0.486	0.403
Aomori	0.57	0.538	0.532	0.384
Iwate	0.521	0.472	0.479	0.458
Miyagi	0.474	0.465	0.469	0.330
Akita	0.405	0.369	0.361	0.325
Yamagata	0.369	0.363	0.370	0.328
Fukushima	0.503	0.468	0.464	0.421
Ibaraki	0.497	0.506	0.512	0.402
Tochigi	0.456	0.421	0.428	0.364
Gumma	0.591	0.514	0.504	0.331
Saitama	0.480	0.445	0.443	0.383
Chiba	0.479	0.465	0.463	0.384
Tokyo	0.377	0.354	0.353	0.289
Kanagawa	0.374	0.353	0.341	0.278
Niigata	0.542	0.470	0.460	0.388
Toyama	0.320	0.319	0.303	0.309
Ishikawa	0.369	0.354	0.354	0.305
Fukui	0.430	0.431	0.421	0.308
Yamanashi	0.546	0.530	0.538	0.461
Nagano	0.407	0.364	0.348	0.286
Gifu	0.347	0.349	0.348	0.282
Shizuoka	0.325	0.319	0.278	0.270
Aichi	0.400	0.344	0.335	0.233
Mie	0.386	0.371	0.377	0.279
Shiga	0.426	0.403	0.398	0.298
Kyoto	0.496	0.480	0.479	0.426
Osaka	0.434	0.398	0.399	0.334
Hyogo	0.429	0.406	0.403	0.352
Nara	0.387	0.375	0.382	0.319
Wakayama	0.469	0.438	0.426	0.405
Tottori	0.354	0.327	0.327	0.251
Shimane	0.506	0.461	0.375	0.316
Okayama	0.542	0.518	0.513	0.404
Hiroshima	0.462	0.448	0.456	0.344
Yamaguchi	0.485	0.426	0.428	0.316
Tokushima	0.542	0.495	0.501	0.411
Kagawa	0.429	0.441	0.412	0.408
Ehime	0.569	0.546	0.547	0.410
Kochi	0.553	0.537	0.534	0.465
Fukuoka	0.430	0.424	0.423	0.348
Saga	0.530	0.515	0.516	0.351
Nagasaki	0.468	0.467	0.469	0.380
Kumamoto	0.567	0.556	0.558	0.475
Oita	0.587	0.536	0.543	0.392
Miyazaki	0.600	0.576	0.573	0.491
Kagoshima	0.524	0.514	0.519	0.428
Okinawa	0.371	0.369	0.374	0.327

DPC, Diagnosis Procedure Combination.
