# Supplementary material for: Prefectural difference in spontaneous intracerebral hemorrhage incidence in Japan analyzed with publically accessible diagnosis procedure combination data: possibilities and limitations
Source: Epidemiol Health. 2016 Jul 2;38:e2016028. doi: 10.4178/epih.e2016028 (PMC5037357; doi:10.4178/epih.e2016028)
Supplement: Supplementary file 7 [file epih-38-e2016028-app7.pdf]

Appendix 7. Prefectural data of examined factors in comparison model with prefectural sICH incidence on the DPC database in 2014, shown in Appendix 3

Name of Prefecture	Population per 1 km^2 of inhabitable area	Yearly average of temperature	Yearly sunshine hours
Hokkaido	244.6	9.2	1,648
Aomori	412.9	10.5	1,516
Iwate	350.6	10.6	1,671
Miyagi	740.2	12.7	1,880
Akita	328.7	11.9	1,469
Yamagata	399.6	11.9	1,696
Fukushima	460.2	13.4	1,844
Ibaraki	736.1	14.3	2,259
Tochigi	666.1	14.4	2,128
Gumma	862.2	15.2	2,366
Saitama	2,805.7	15.6	2,336
Chiba	1,753.2	16.6	2,135
Tokyo	9,553.6	17.1	2,131
Kanagawa	6,187.3	16.6	2,257
Niigata	517.3	13.8	1,662
Toyama	580.8	14.5	1,786
Ishikawa	834.4	15.0	1,868
Fukui	740.2	14.9	1,740
Yamanashi	889.4	15.4	2,462
Nagano	640.4	12.3	2,130
Gifu	932.2	16.3	2,316
Shizuoka	1,352.1	17.2	2,298
Aichi	2,501.5	16.4	2,355
Mie	896.7	16.5	2,366
Shiga	1,092.1	15.1	2,073
Kyoto	2,222.8	16.2	1,940
Osaka	6,701.4	17.1	2,300
Hyogo	2,002.5	17.0	2,255
Nara	1,624.3	15.3	2,004
Wakayama	893.3	17.0	2,282
Tottori	634.6	15.5	1,842
Shimane	545.0	15.4	1,843
Okayama	866.5	16.4	2,214
Hiroshima	1,239.8	16.6	2,249
Yamaguchi	827.4	15.8	2,041
Tokushima	751.8	16.8	2,327
Kagawa	982.1	16.8	2,288
Ehime	842.7	16.8	2,208
Kochi	641.9	17.3	2,373
Fukuoka	1,833.7	17.7	2,059
Saga	630.2	17.1	2,113
Nagasaki	854.7	17.5	2,018
Kumamoto	659.2	17.2	2,197
Oita	674.7	16.9	2,186
Miyazaki	606.8	17.9	2,411
Kagoshima	513.7	18.9	2,183
Okinawa	1,211.4	23.3	1,809

(continued)

Name of Prefecture	Yearly precipitation	Prefectural income per person	Prefectural alcohol consumption per adult person
Hokkaido	1,347	2,475	89.73
Aomori	1,664	2,333	95.75
Iwate	1,643	2,359	91.06
Miyagi	1,112	2,461	88.36
Akita	2,373	2,319	94.65
Yamagata	1,347	2,403	83.07
Fukushima	1,202	2,324	82.28
Ibaraki	1,338	3,044	68.49
Tochigi	1,505	2,955	68.83
Gumma	999	2,890	71.35
Saitama	1,251	2,785	70.37
Chiba	1,447	2,820	72.52
Tokyo	1,614	4,373	109.37
Kanagawa	1,517	2,926	74.73
Niigata	2,327	2,668	94.68
Toyama	2,863	3,055	84.00
Ishikawa	3,318	2,744	81.66
Fukui	2,726	2,841	79.14
Yamanashi	939	2,779	75.97
Nagano	1,138	2,730	77.53
Gifu	1,876	2,657	67.35
Shizuoka	1,822	3,162	71.48
Aichi	1,464	3,105	74.35
Mie	1,394	2,735	70.09
Shiga	1,492	3,072	63.52
Kyoto	1,451	2,865	84.98
Osaka	1,418	2,920	94.12
Hyogo	1,298	2,585	80.05
Nara	1,506	2,388	65.41
Wakayama	1,370	2,655	81.88
Tottori	2,048	2,232	85.08
Shimane	2,035	2,382	83.56
Okayama	1,341	2,693	70.60
Hiroshima	1,821	3,030	84.64
Yamaguchi	2,267	2,864	78.50
Tokushima	1,949	2,698	73.55
Kagawa	1,537	2,790	76.36
Ehime	1,623	2,673	78.70
Kochi	2,327	2,199	98.91
Fukuoka	1,802	2,778	81.16
Saga	2,012	2,399	74.60
Nagasaki	1,684	2,351	76.36
Kumamoto	1,975	2,399	84.23
Oita	1,506	2,488	78.95
Miyazaki	2,080	2,208	94.96
Kagoshima	1,778	2,431	91.48
Okinawa	2,071	2,018	97.10

sICH, spontaneous intracerebral hemorrhage; DPC, Diagnosis Procedure Combination.
